# The German Center for Child and Adolescent Health – A new structure for translational research in pediatrics shaping the health of children today and future generations

**DOI:** 10.1186/s40348-025-00198-w

**Published:** 2025-08-30

**Authors:** Klaus-Michael Debatin, Jutta Gärtner, Christoph Klein, Antje Körner, Marcus A. Mall, Ania C. Muntau, Neeltje van den Berg

**Affiliations:** 1 Department of Pediatrics and Adolescent Medicine, University Medical Center Ulm, Eythstrasse 24, 89075 Ulm, Germany; 2 Department of Pediatrics and Adolescent Medicine, University Medical Center Göttingen, Göttingen, Germany; 3 Department of Pediatrics, Dr. von Hauner Children’s Hospital, University Hospital, Ludwig-Maximilians-University Munich, Lindwurmstr. 4, 80337 Munich, Germany; 4 Center for Pediatric Research, Hospital for Children and Adolescents, Medical Faculty, Leipzig University, Leipzig, Germany; 5 Department of Pediatric Respiratory Medicine, Immunology and Critical Care Medicine, Charité - Universitätsmedizin Berlin, Berlin, Germany; 6 University Children’s Hospital, University Medical Center Hamburg-Eppendorf, Hamburg, Germany; 7 Department of Epidemiology of Health Care and Community Health, Institute for Community Medicine University Medicine Greifswald, Greifswald, Germany; 8 German Center for Child and Adolescent Health (DZKJ), Göttingen, Germany

## Abstract

The new German Center for Child and Adolescent Health (DZKJ) founded as part of the German Centers for Health Research provides an unprecedented and unique opportunity for internationally outstanding research that contributes to the health and well-being of children and adolescents by creating a sustainable, multidisciplinary translational research center with a wide spectrum of clinical and scientific disciplines. The DZKJ attracts and motivates some of the best basic and clinical scientists in Germany inside and outside the field of pediatrics to jointly dedicate their research and creativity to unravelling the causes of both common and rare diseases and to developing innovative therapies and prevention strategies. All DZKJ partner sites will join forces for a pivotal, networked lighthouse for clinical and translational science in pediatrics in Germany and beyond.

From the perinatal period to adulthood, the health of children and adolescents is threatened by a variety of intrinsic and extrinsic factors leading to rare or common diseases. Consequently, diagnosis and treatment of pediatric diseases requires a specific focus and specialized medicine for this age group [1, 2]. Childhood and adolescence also set the stage for the health status later in life. The early years and early decades are particularly susceptible to disturbances leading to dysregulations of key systems of the body as the basis for the course of many common diseases. Also, the vast majority of the more than 7,000 rare diseases of which 80% are genetic in origin have a disease manifestation in early years [3]. Thus, pediatrics and pediatric research are challenged not only by the complexity of treating common and rare diseases but also by the responsibility to provide the basis for life-long health of the individual. This includes the challenge to provide the best health care for this vulnerable population as well as early detection and prevention of potential threats to health following Article 24 of the UN Convention on the Rights of a Child which states that parties recognize the right of the child to the enjoyment of the highest attainable standard of health and to facilities for the treatment of illness and rehabilitation of health.

Recognizing the particular vulnerabilities of its young citizens and their right to the highest attainable standard of health, the German government has established a new national research center, the German Center for Child and Adolescent Health (Deutsches Zentrum für Kinder- und Jugendgesundheit, DZKJ) [4]. Visionary concepts paired with scientific excellence were the basis for a competitive international scientific review and selection process. Seven sites were selected and subsequently joined forces to develop the concept of a united, interdisciplinary DZKJ as a national center to promote excellence in pediatric research for the decades to come (Fig. 1). The vision and mission of the DZKJ are reflected by focus areas that address diseases in overarching aspects critical for the health of children and adolescents and their development [5].

**Fig. 1:**
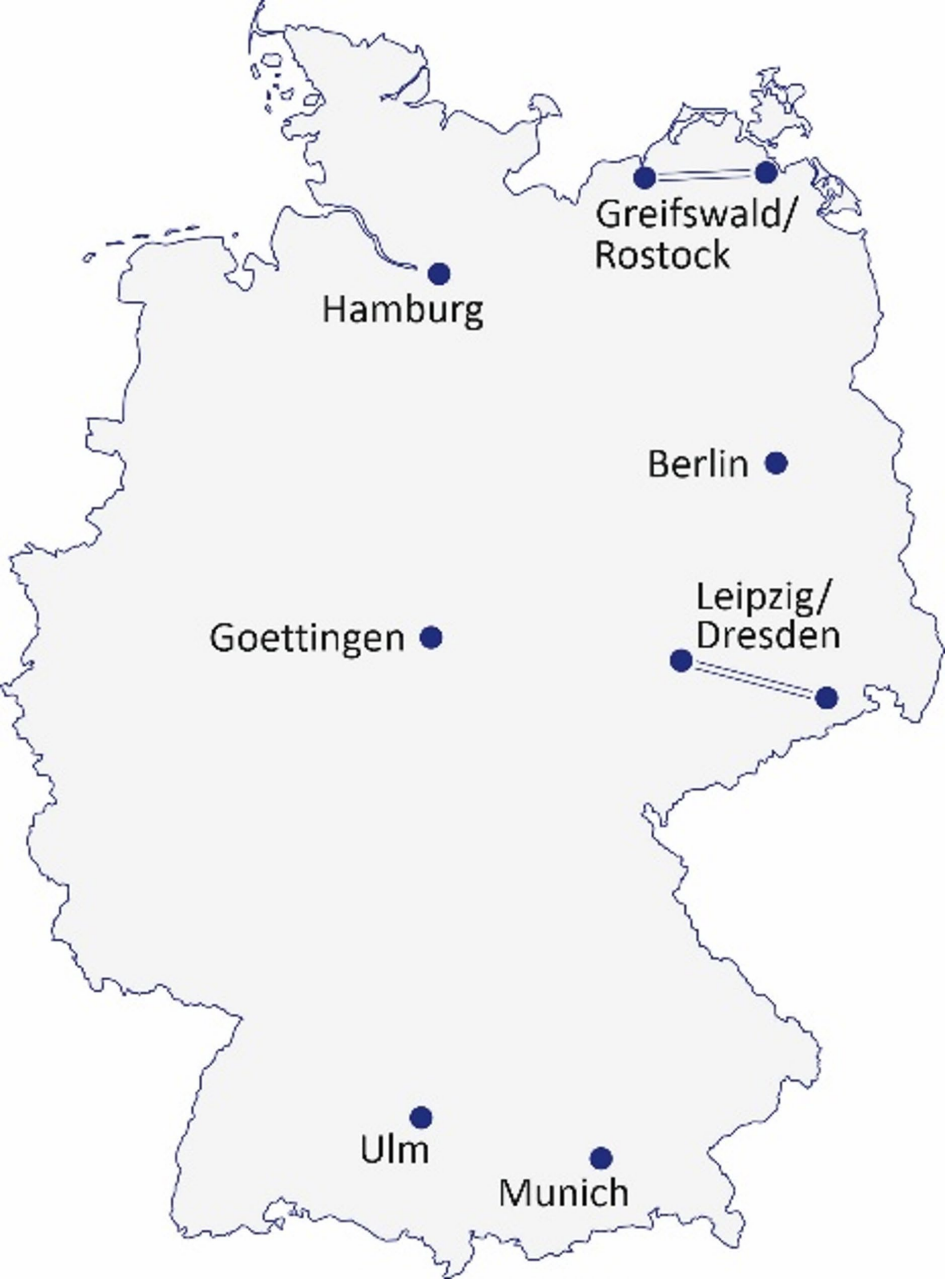
**Map of the seven DZKJ partner sites**

The DZKJ will address the challenge of a cross-disciplinary and disease-overarching approach, applied to child and adolescent health. Moreover, it will include the whole life trajectories and holistic perspectives of human development from the prenatal stage to young adults. The DZKJ aims to accelerate diagnostic and therapeutic innovation, harmonize diagnostic and therapeutic programs, establish a DZKJ-wide population-based pediatric cohort, establish a DZKJ-wide pediatric data management structure, ensure nationwide access to diagnostic and therapeutic innovations, and establish preventive measures that protect children and adolescents from chronic disability. Even though many of the common diseases are diagnosed in adult age, the pathogenesis and manifestation start much earlier and already in childhood. Nevertheless, children and adolescents do not adequately benefit from the scientific and clinical advance. It is the ambition of the DZKJ to overcome this research and treatment gap. By this, the DZKJ will serve healthy and chronically ill children and adolescents, as well as parents, families, the scientific community, and the society.

Addressing the so far unmet needs of children and adolescents in the German research system, the DZKJ has gathered a powerful team of basic and clinical scientist and health services researchers in an ambitioned translational research program projected for the next 10 to 15 years. Its aspiring translational research program will fundamentally contribute to and change knowledge of human development and pediatric diseases and will include novel targeted and precision medicine driven diagnostic and therapeutic strategies for children and adolescents with severe diseases. The research portfolio of the DZKJ is structured in seven defined and interconnected research areas from rare genetic diseases to development of the immune system, immunity, inflammation and infection to CNS development and neurological diseases, to obesity and metabolism, early determinants of health and disease to community medicine as the bridge to bring innovations to the patient and finally with a program of psychosocial and mental health research as an important bridge to the also newly established German Center for Mental Health (Fig. 2) [6].

**Fig. 2:**
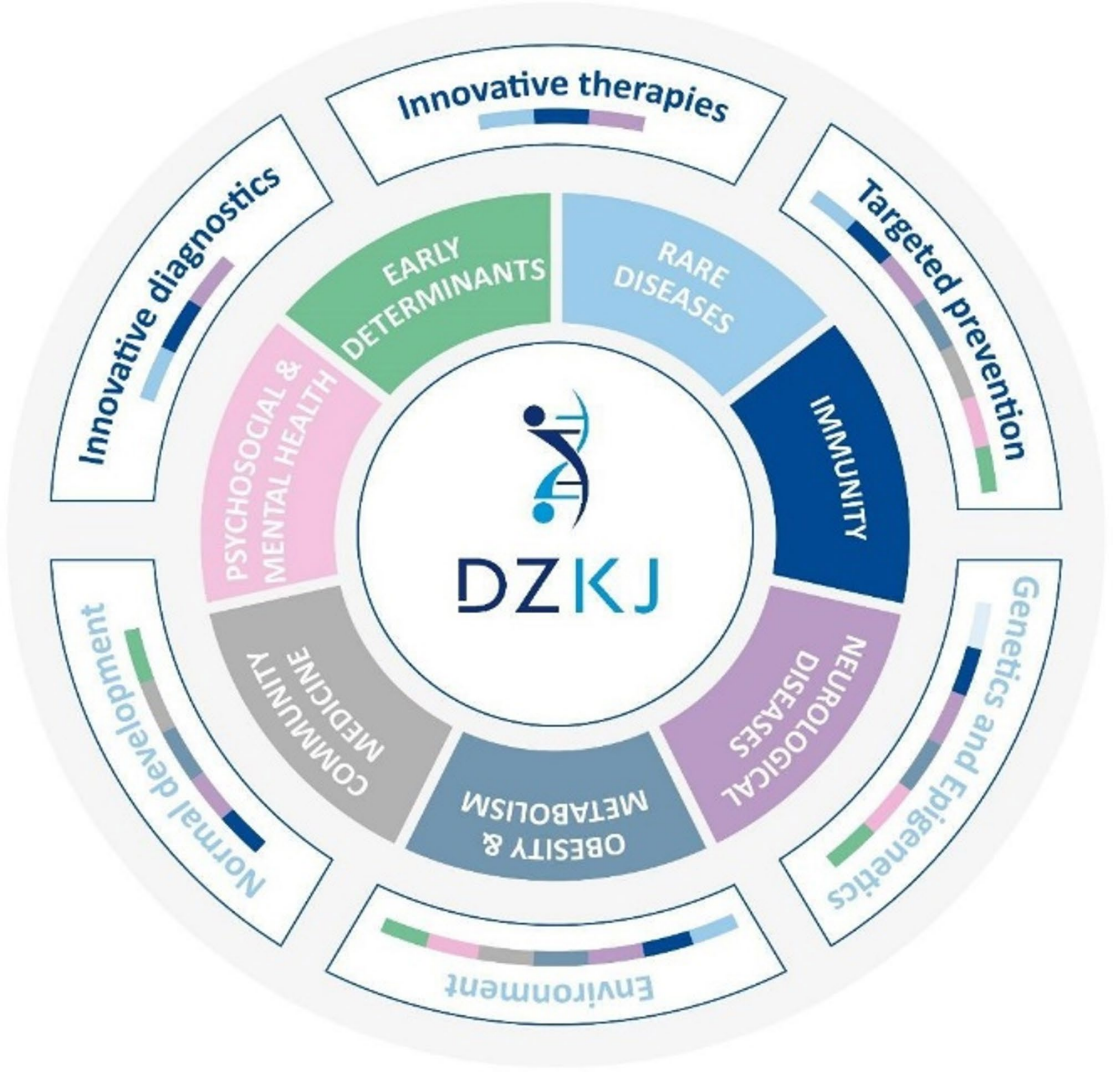
**Interdependencies of research elements in the seven central research areas**. The DZKJ aims to promote child and adolescent health by accelerating breakthroughs in basic research, diagnosis, treatment, and prevention of diseases. It focuses on seven main research areas shown in colored boxes. The outer circle shows bridging research themes, each covered by several main research areas (depicted in color)

Specifically, the DZKJ will focus on major unmet medical needs for children and adolescents that urgently need to be addressed at national and international levels:

- For the vast majority of the more than 7,000 rare diseases there is a need for rapid precision diagnostics. Effective targeted or even curative treatment is only available for a small subset of these patients. Rare diseases usually have a genetic cause and become manifest in childhood and adolescence. Three out of ten children with a rare disease die before the age of five [7]. Thus, the center shall provide strategies for treating rare genetic diseases based on understanding based on molecular pathomechanisms.
- Dysregulated immunity sets the stage for susceptibility to infection, allergies, autoimmunity, and chronic inflammation. Many of these severe chronic diseases such as type 1 diabetes, juvenile rheumatoid arthritis, celiac disease, and inflammatory bowel disease start in early childhood, have limited therapeutic options, and may lead to substantial lifelong morbidity [8].
- Diseases of the central nervous system (CNS) lead to severe long-lasting consequences for the patients, their families and society. Understanding the basis of normal development of the CNS and its aberration is key to preventing and treating of e.g., genetically determined retardation syndromes, neurodegeneration, and autoinflammation [9].
- Currently, the greatest life-long threat to the health of children arises from non-communicable diseases. Particularly, the prevalence of overweight and obesity and its ensuing comorbidities, increasing from > 20% in children/adolescents up to > 40% in the adult population [10]calls for immediate action to understand and tackle the complex origin of obesity, the principles of a disturbed metabolism and to the development of targeted prevention and innovative treatment programs including the improvement of access to these programs.
- Multiple environmental exposures including specific socio-environmental factors may affect the physical, mental and development and function of all human organs and prime the susceptibility and manifestation of developmental disabilities and chronic common diseases in childhood, adolescence and beyond [11].
- Psychosocial determinants are key drivers for the physical and mental health status and the quality of life of children and adolescents and their families, which often receive insufficient attention in the case of children and adolescents with severe somatic diseases [12].
- Finally, every child must have access to medical innovations; scientific and clinical breakthroughs have to be translated into medical practice so that the whole population, including vulnerable groups, will benefit at all levels of medical care and social participation [13].

The research areas are supported by a network of infrastructure and expertise in biobanking, multi-omics approaches, IT innovation involving internationally renowned research institutions which participate in the overall consortium. The DZKJ follows a holistic approach and will establish a new population-based pediatric cohort with modules of both healthy and chronically ill children and adolescents across the entire age span from fetal life to adolescence. While this will be based on already existing large cohorts at the partner sites, the new DZKJ cohort will be established as a longitudinal study across age ranges and regional characteristics of the population.

The DZKJ is committed to initiate and accelerate clinical trials involving novel therapeutic approaches and targeted therapies in particular for the diseases addressed by the research areas open for children in Germany to participate.

As a unique enterprise, the DZKJ will integrate children and young people into the center’s advisory boards, establishing the first national framework for the empowerment of young people in research. A new focus on patient, parent, public involvement will be relevant in all phases and programs of the research network.

In the DZKJ academy we will train the next generation of health professionals and researchers. Thus, new leaders in academic medicine in Germany and beyond will design the next era of translational research in child and adolescent health and medical care.

Finally, given the fact that the specific needs for child and adolescent health including medical care are currently only partially addressed, we expect that the research programs of the DZKJ will also provide a unique framework to generate novel intellectual property leading to entrepreneurship, spin-offs etc. with ultimately also economic value also for the society.

The DZKJ is the youngest member of the Alliance of German Health Research Centers (DZG) [14]. Previously founded centers focus on Cancer (DKTK), Infectious Diseases (DZIF), Lung Diseases (DZL), Neurodegenerative Disorders (DZNE), Diabetes (DZD), Cardiovascular Diseases (DZHK), and Mental Disorders (DZPG). The DZKJ will synergistically complement the existing DZGs and develop joint research platforms, especially for children and adolescents. The DZKJ aims to invite and support the best scientists to put their talents and scientific excellence at the service of sick children and adolescents and their families. Furthermore, the DZKJ will serve as a national hub to build global alliances. The researchers and the Federal Ministry of Education and Research (BMBF) are united in their vision to build an internationally visible beacon of translational science to empower children, adolescents and their families and to contribute to the development of a new era of precision medicine in diagnostics, therapeutics and prevention. With its program, the DZKJ is to become an internationally visible center for pediatric research.
